# A neuronal network model for context-dependence of pitch change perception

**DOI:** 10.3389/fncom.2015.00101

**Published:** 2015-08-06

**Authors:** Chengcheng Huang, Bernhard Englitz, Shihab Shamma, John Rinzel

**Affiliations:** ^1^Courant Institute of Mathematical Sciences, New York UniversityNew York, NY, USA; ^2^Electrical and Computer Engineering Department, Institute for Systems Research, University of MarylandCollege Park, MD, USA; ^3^Laboratoire des Systèmes Perceptifs, Equipe Audition, Ecole Normale SuperieureParis, France; ^4^Department of Neurophysiology, Donders Institute, Radboud UniversityNijmegen, Netherlands; ^5^Donders Center for Neuroscience, Donders InstituteNijmegen, Netherlands; ^6^Center for Neural Science, New York UniversityNew York, NY, USA

**Keywords:** auditory illusion, adaptation, neuromechanistic modeling, Shepard tone, context

## Abstract

Many natural stimuli have perceptual ambiguities that can be cognitively resolved by the surrounding context. In audition, preceding context can bias the perception of speech and non-speech stimuli. Here, we develop a neuronal network model that can account for how context affects the perception of pitch change between a pair of successive complex tones. We focus especially on an ambiguous comparison—listeners experience opposite percepts (either ascending or descending) for an ambiguous tone pair depending on the spectral location of preceding context tones. We developed a recurrent, firing-rate network model, which detects frequency-change-direction of successively played stimuli and successfully accounts for the context-dependent perception demonstrated in behavioral experiments. The model consists of two tonotopically organized, excitatory populations, *E*_up_ and *E*_down_, that respond preferentially to ascending or descending stimuli in pitch, respectively. These preferences are generated by an inhibitory population that provides inhibition asymmetric in frequency to the two populations; context dependence arises from slow facilitation of inhibition. We show that contextual influence depends on the spectral distribution of preceding tones and the tuning width of inhibitory neurons. Further, we demonstrate, using phase-space analysis, how the facilitated inhibition from previous stimuli and the waning inhibition from the just-preceding tone shape the competition between the *E*_up_ and *E*_down_ populations. In sum, our model accounts for contextual influences on the pitch change perception of an ambiguous tone pair by introducing a novel decoding strategy based on direction-selective units. The model's network architecture and slow facilitating inhibition emerge as predictions of neuronal mechanisms for these perceptual dynamics. Since the model structure does not depend on the specific stimuli, we show that it generalizes to other contextual effects and stimulus types.

## Introduction

The auditory world is encoded in a time-varying pressure field with a mix of multiple acoustic sources, each characterized by its spectral and temporal properties. Listeners are continuously faced with the challenge to segregate auditory sources, such as ongoing music and the voice of a person speaking nearby. This task of segregating and extracting relevant information from the composite acoustic signal is known as auditory scene analysis (Bregman, 1994). The preceding context of stimuli strongly influences the way we process the current sound, since the recent history of each source is highly correlated with what comes next. Making use of the past history enables us to segregate present stimuli and bind them with the past to form a continuous acoustic entity, such as a melody or a word. However, the computational mechanisms underlying this dependence on stimulus history are not completely understood. In the present work, we develop a neuronal network model to explain the context effects on directional perception (i.e., ascending vs. descending steps in pitch), one of the basic relationships for binding successive tones. The model draws inspiration from recent work (Englitz et al., 2013) about the influence of preceding stimuli on directional perception of artificially designed ambiguous tone pairs.

**Figure 1:**
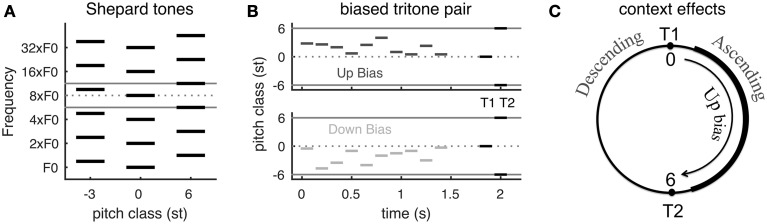
**The Psychophysical experiment paradigm and summary of behavioral results**. **(A)** Schematic of Shepard tones (details see Materials and Methods). A Shepard tone consists of multiple octave-spaced pure tones. Due to the periodic spectral structure of Shepard tones, we can represent each tone by its pitch class within one octave (between the two gray lines). A tritone pair is two Shepard tones separated by a half-octave, for example the tones at pitch classes 0 st (middle) and 6 st (right). **(B)** Stimuli examples in tritone comparison with preceding bias tones. The bias tones are randomly sampled in the region either above (*Up bias*) or below (*Down bias*) the first test tone (T_1_). T_1_ and T_2_ is a tritone pair, separated by a half-octave (6 st). **(C)** Steps of 0–6 st from T_1_ (tones at the right half of the pitch class circle) are perceived as ascending while steps of -6–0 st (tones at the left half circle) are perceived as descending (Shepard, 1964; Chambers and Pressnitzer, 2014). *Up bias* tones bias the perception of the ambiguous tritone pair (T_1_ and T_2_) toward ascending while *Down bias* tones bias toward descending (Englitz et al., 2013, Figure 18.1D, see Supplementary Material for audio demonstrations). [**A,B** are modified from Englitz et al. (2013). (Figures 18.1A,C)].

The psychophysical experiments (Repp, 1997; Englitz et al., 2013) adopt Shepard tones, each of which consists of multiple simultaneous octave-spaced pure tones (Figure 1A). A Shepard tone with many frequency components is approximately spectrally periodic. Shepard tones are famous for being used to create the auditory illusion of an ever-ascending sequence of tones. This is done by incrementing the *pitch class* (PC), note name in music, by 1 semitone (st) at a time, although the sequence repeats itself for every 12 tones due to the spectral periodicity (1 octave is 12 st) (Shepard, 1964). When two Shepard tones are separated by a half-octave (tritone) (e.g., tones at PC = 0 and 6 st in Figure 1A), the pitch change direction is ambiguous and the directional percept of the same tritone pair varies among subjects (Deutsch, 1986, 1991; Deutsch et al., 1990). Strong hysteresis effects have been shown for tritone pairs (Giangrande et al., 2003; Chambers and Pressnitzer, 2014), suggesting that directional percepts of tritone pairs are very susceptible to preceding stimuli, i.e., context. (Repp, 1997 Experiment 3) found that a single Shepard tone before a tritone pair influences the perceived pitch change direction. A few preceding Shepard tones with PC between the tritone pair can strongly bias the perception toward the direction from the first (T_1_) to the second tone (T_2_)—ascending if the sequence is within the half-octave interval above T_1_, and vice versa if below T_1_(Englitz et al., 2013, see Figure 18.1D; Chambers and Pressnitzer, 2011) (Figures 1B,C; for details see Materials and Methods, see Supplementary Material for audio demonstrations).

The directional percept of a Shepard tone pair depends on the spectral interval from T_1_ to T_2_ on a pitch class circle: ascending if the interval is less than 6 st and descending if more than 6 st (equivalently the interval from T_2_ to T_1_ is less than 6 st) (Shepard, 1964; Chambers and Pressnitzer, 2014) (Figure 1C). Such dependence is referred to as the proximity principle by Shepard (1964). A neural computation for such a relationship, however, is not straightforward, since the spectra of Shepard tones are interleaved. Although the proximity principle implies a shorter distance between the tritone pair across the biasing region after the preceding tones, a recent neural decoding approach demonstrates a slightly larger distance between population representations of pitch across the biasing region in primary auditory cortex of awake ferrets (Englitz et al., 2013) (Figure 1C). The paradigm used in the referred study was identical to the present paradigm, and evaluated the influence of preceding biasing tones on the estimated pitch of the components of the Shepard tone. While the perceptual results suggest a reduction of the distance of these components, an increase in distance was observed, due to local adaptation of neural responses. This suggests that such a *pitch-based* algorithm is not adequate to explain the biasing effects. This inadequacy and our goal to develop a neuromechanistic model motivated the current work on *pitch-change detection* as underlying the frequency comparison of complex tones and context effects on the comparison.

Direction-selective units have been suggested in previous studies of auditory perception. The existence of frequency shift detectors was proposed by Demany and Ramos (2005) when they found that subjects could perceive an upward or downward pitch shift without recognizing individual components within a chord. Physiological evidence for direction-selective neurons to frequency-modulated sweeps has been found along the auditory pathway: in inferior colliculus (Nelson et al., 1966; Gordon and O'Neill, 1998; Fuzessery et al., 2006), auditory thalamus (O'Neill and Brimijoin, 2002) and the primary auditory cortex (Suga, 1965; Mendelson and Cynader, 1985; Zhang et al., 2003). However, these studies involved sweeps at much faster time scales (70 oct/s) than in the experiments with Shepard tones (see Discussion). Direction selectivity has been implicated in a theoretical study of a delayed match-to-sample auditory task (Husain et al., 2004), although without consideration for context effects.

Our model provides the first neuromechanistic framework to account for context effects on pitch change perception, with an application to the ambiguous tritone comparison. It makes a local comparison of frequency components in successive tone pairs using asymmetric inhibition. This inhibition creates a dynamic competition between two direction-selective excitatory populations, *E*_*up*_ and *E*_*down*_. Comparisons of Shepard tone pairs using the model agree with those in psychophysical studies. A novel adaptation mechanism, facilitation of inhibitory synapses, is incorporated to account for the biasing effects. The slowly facilitated inhibitory synapses in the stimulated region provide a spectral representation of the past stimuli and shape the competition between *E*_*up*_ and *E*_*down*_ populations according to relative positions. The biasing effects gradually accumulate with the number of bias tones with the same rate as in human studies. Further, we demonstrate the model's generality by showing that it can detect frequency shifts for stimuli that are not spectrally periodic. Lastly, we use phase-space analysis to investigate the biasing mechanisms in a simplified winner-take-all model.

## Materials and methods

### Network model

#### Stimuli

The stimuli in the present model are simulated sounds. Each sound is a sequence of complex tones, so-called Shepard tones (Shepard, 1964) (Figure 1A). A Shepard tone is a stack of synchronous octave-spaced pure tones. Each Shepard tone has a pure tone frequency, ranging from arbitrarily low to arbitrarily high frequencies (if physically realized, the human hearing range would naturally limit this range). In the present study each frequency component within a Shepard tone is assumed to have the same amplitude, i.e., leading to a flat spectrum envelope. Due to this regular structure in frequency, a Shepard tone shifted by one octave is mapped onto physically the same Shepard tone. The stimulus space of Shepard tones therefore has a circular structure (akin to oriented bars in the visual system). Consequently we can represent all Shepard tones conveniently within one octave, where each Shepard tone is represented by its pitch class *x* within this octave, ranging within [0, 12] semitones, corresponding to one full octave. This transformation corresponds to a group-theoretic modulo operation and can be performed without loss of generality.

In the model, we represent a Shepard tone of pitch class *x*_0_ as a Gaussian function centered at *x*_0_ with width of σ_*in*_ = 0.1 octaves (Equation 1). In the temporal domain each Shepard tone is gated by a cosine ramp at its beginning and end with a time constant τ_*r*_ = 5 ms. The onset/offset ramps are often utilized to prevent a clicking sound in auditory psychophysics. The tone durations were 100 ms unless noted otherwise.

(1) $$Input(x,t)=\exp\left(-\frac{{\left(x-x_{0}\right)}^{2}}{\sigma_{in}^{2}}\right)ramp(t-t_{1})ramp(t_{2}-t),$$

where *ramp*(*t*) = ((cos(π(*t*/τ_*r*_ + 1)) + 1)2)^2^if *t* < τ_*r*_ and 1 otherwise.

A tritone pair is two Shepard tones separated by a half-octave, such as tones at 0 st (middle) and 6 st (right) shown in Figure 1A. In simulated experiments of a tritone comparison with bias tones (**Figures 5**, **6**), *N*_*bias*_ Shepard tones are randomly sampled either within +6 st (*Up bias*) or -6 st (*Down bias*) step from T_1_ (Figure 1B). *Up bias* tones lead to an ascending percept for the following tritone test pair, while *Down bias* tones lead to a descending percept (Englitz et al., 2013). The tone duration is 100 ms and inter-tone interval is 50 ms; the gap between bias tones and tritone pair is 500 ms. Audio demonstrations of context effects on a tritone pair can be found in Supplementary Material.

#### Model specification

**Figure 2:**
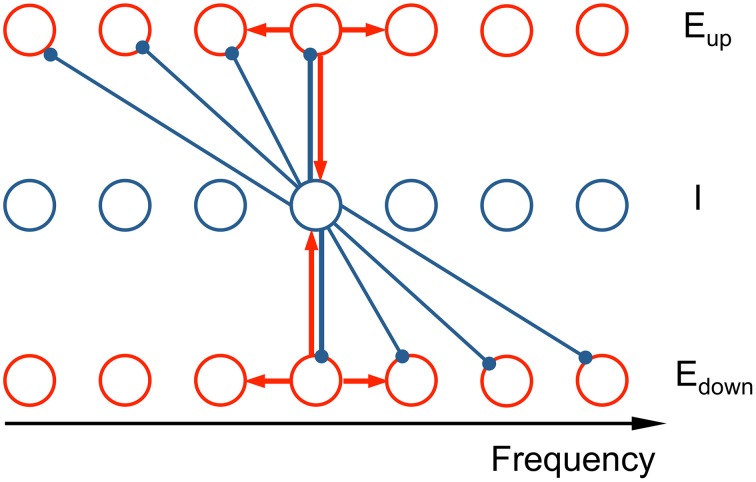
**Schematic of the connectivity in the neuronal network model**. The network model consists of two excitatory populations (*E*_up_ and *E*_down_) and an inhibitory population (*I*), tonotopically organized. The asymmetric inhibitory feedback leads to an ascending/descending frequency change preference for the *E*_up_ and *E*_down_ populations, respectively. Each unit is a local subpopulation, positioned at its characteristic frequency (CF). Activity of each unit is described by a firing rate, whose dynamics are governed by the differential equations (see Equation 2 in Materials and Methods). Red arrows signify recurrent excitation and blue arrows inhibition. The subset of the connections shown illustrates the architecture's qualitative nature: the synaptic footprints from *E* to *E* and from *E* to *I* are narrow and symmetric; from *I* to *E* the footprint is broad and asymmetric.

Our network model consists of three tonotopically organized subpopulations: two excitatory (*E*) populations that drive a common inhibitory (*I*) population and the latter provides recurrent inhibition but with oppositely directed asymmetric projective fields (ω_*up*_, ω_*down*_) (see schematic in Figure 2). The model describes the firing rate dynamics of three populations as a continuum in frequency, where each location in frequency corresponds to a neuron with this location as its characteristic frequency (CF).

The normalized firing rates of the two excitatory populations, *E*_up_ and *E*_down_, and the inhibitory populations are, respectively, *r*_*up*_(*x, t*), *r*_*down*_(*x, t*), and *r*_*I*_(*x, t*) with CF *x* and at time *t*. The excitatory populations exhibit direction selectivity in their response to steps in stimulus frequency. This selectivity is implemented via the connectivity structure of the inhibitory neurons: Inhibitory neurons inhibit lower frequency *E*_up_ units and higher frequency *E*_down_ units, thus making them selective to ascending and descending frequency change respectively (Ye et al., 2010). The differential equations of firing rates are in the spirit of the classical Wilson-Cowan approach (Wilson and Cowan, 1972, 1973). Due to the spectral periodic structure of Shepard tones (consists of octave-spaced pure tones), we need to consider only one octave instead of the entire frequency range. This reduction is equivalent to the full model with periodic boundary conditions. In this way, the model uses dimensionless firing rates and frequencies.

To model the long-term effects of previous tones, we include slow facilitation, *F*(*x, t*), of inhibitory synaptic drive, which accumulates when an inhibitory neuron is activated (Ermentrout and Terman, 2010, see Section 7.2). Non-uniform *F*(*x, t*) gives different inhibitory currents on *E*_*up*_ and *E*_*down*_ populations, thus biasing the perception of a tritone comparison.

The equations of our model are as follows:

(2) $$\{\begin{array}{l} \tau_{e}\frac{\text{d}r_{up}(x,t)}{\text{d}t}=-r_{up}(x,t)+S_{e}(h_{ee}^{up}(x,t)-h_{ie}^{up}(x,t)\\ \;+\gamma_{e}Input(x,t))\\ \tau_{e}\frac{\text{d}r_{down}(x,t)}{\text{d}t}=-r_{down}(x,t)+S_{e}(h_{ee}^{down}(x,t)-h_{ie}^{down}(x,t)\\ \;+\gamma_{e}Input(x,t))\\ \tau_{i}\frac{\text{d}r_{I}(x,t)}{\text{d}t}=-r_{I}(x,t)+S_{i}(h_{ei}^{up}(x,t)+h_{ei}^{down}(x,t)\\ \;+\gamma_{i}Input(x,t))\\ \frac{\text{d}F(x,t)}{\text{d}t}=-\frac{F(x,t)}{\tau_{\text{fd}}}+\frac{r_{I}(x,t)\left(1-F(x,t)\right)}{\tau_{\text{fr}}} \end{array}$$

where *S*_*e*_ and *S*_*i*_ are sigmoidal functions representing the steady state input-output relation of neurons (on average) and firing activity is normalized to the range: 0 ≤ *x* ≤ 1.

(3) $$S_{\beta }(x)=s_{0}\left(\frac{1}{1+\exp\left(\left(\theta_{\beta }-x\right)k_{\beta }\right)}-x_{0}\right),\beta =e,i$$

with $x_{0}=\frac{1}{1+\exp\left(\theta_{\beta }/k_{\beta }\right)},s_{0}=\frac{1}{1-x_{0}}$, θ_*e*_ = 0.5, *k*_*e*_ = 0.1, θ_*i*_ = 0.3, *k*_*i*_ = 0.2. The time constants of excitatory and inhibitory populations are τ_*e*_ = 20 ms, τ_*i*_ = 30 ms. The facilitation level, *F*, is a slow variable with rise time constant τ_*fr*_ = 100 ms and decay time constant τ_*fd*_ = 2000 ms. The synaptic drive that a unit at *x* receives from another unit at *x* − *y* is the firing rate of the presynaptic unit *r*(*x* − *y, t*), weighted with synaptic strength ω(*y*) which depends on the distance *y* between CF's of presynaptic neuron and post-synaptic neuron. The total synaptic current *h*(*x, t*) is a convolution of firing rates of presynaptic population and synaptic weight function.

(4) $$\begin{array}{l} h_{ie}^{\alpha }(x,t)=a_{ie}\int \omega_{\alpha }(y)\left(1+\gamma_{f}F(x-y,t)\right)r_{I}(x-y,t)dy,\\ h_{e\beta }^{\alpha }(x,t)=a_{e\beta }\int \omega_{e\beta }(y)r_{\alpha }(x-y,t)dy,\left(\alpha =up,down,\beta =e,i\right) \end{array}$$

The overall synaptic strengths were set to *a*_*ee*_ = 0.7, *a*_*ei*_ = 2, and *a*_*ie*_ = 1.5. Values for other parameters are γ_*f*_ = 2, γ_*e*_ = 0.6, and γ_*i*_ = 0.2.

#### Synaptic footprints

The connectivity structure between the neural populations is governed by the set of synaptic weight functions ω_*ee*_(excitatory to excitatory), ω_*ei*_ (excitatory to inhibitory), ω_*up*_ (inhibitory to excitatory *up*-cells) and ω_*down*_ (inhibitory to excitatory *down*-cells), which are all normalized to unit area.

(5) $$\begin{array}{l} \omega_{ee}(x)=z_{1}\exp\left(-\frac{x^{2}}{\sigma_{ee}^{2}}\right),\omega_{up}(x)=\{\begin{array}{cc} 0 & ,x>0\\ z_{3}\exp\left(-\left|x\right|/\sigma_{ie}^{up}\right) & ,x\leq 0 \end{array},\\ \omega_{ei}(x)=z_{2}\exp\left(-\frac{x^{2}}{\sigma_{ei}^{2}}\right),\omega_{down}(x)=\{\begin{array}{cc} z_{4}\exp\left(-\left|x\right|/\sigma_{ie}^{down}\right) & ,x\geq 0\\ 0 & ,x<0 \end{array} \end{array}$$

where *z*_*i*_ are normalization factors and σ_*ee*_ = 0.02, σ_*ei*_ = 0.08, and σ_*ie*_ = 0.3 octaves (Ye et al., 2010; Kuo and Wu, 2012). σ_*ee*_ is chosen small in comparison to σ_*ei*_ such that the effect of recurrent excitation remains localized. The width of the synaptic connectivity from excitatory to inhibitory cells, σ_*ei*_, is larger (than σ_*ee*_) so that the inhibitory population inherits broader responses to tones, which constrains activity of the *E* population from spreading and thereby prevents propagation of activity and controls over-excitation. σ_*ie*_ is chosen large so that the model can detect frequency change of more than 0.5 octaves. In simulations with a broad tuning width of *I* units (Section Biasing Effects Depend on the Spectral Distribution of Bias Tones and Tuning Width of I units, **Figure 6**), σ_*ee*_ = 0.05, σ_*ei*_ = 0.2 octaves and *a*_*ee*_ = 1.5, and the values of other parameters are unchanged.

#### Decision criteria

Decisions are made based on the mean activity difference (*D*) of *E*_*up*_ and *E*_*down*_ during current tone, normalized by the sum of their activities to range between -1 and 1. To relate to human perception, *D* > 0 is interpreted as an ascending percept, *D* < 0 as a descending percept.

(6) $$D=\left(r_{up}-r_{down}\right)/\left(r_{up}+r_{down}\right),$$

$$r_{\alpha }=\frac{1}{T}\underset{\{t:\text{ current tone\}}}{\int \int }r_{\alpha }(x,t)dxdt,\alpha =up,down$$

Where *r*_*up*_ and *r*_*down*_ are the mean activities of *E*_*up*_ and *E*_*down*_ populations during the current tone, respectively. *T* is the duration of current tone. As for comparing our model's behavior with experimental observations, we seek qualitative agreement since the psychophysical and neurophysiological literature on the topic is still too limited to justify quantitative comparison.

#### Numerical integration

The frequency domain *x* is discretized into 100 equal-spaced points in [0, 1] with Δ*x* = 0.01 octave. Boundary conditions are periodic. We use an explicit Runge-Kutta method of 4th order accuracy to integrate in time. The time step size is adjusted at each step such that relative error and absolute error are less than 10^−5^.

### 3-variable winner-take-all (WTA) model

To analyze the biasing mechanisms of the context in the network model, we consider an idealized model of three variables without frequency dependence: two excitatory populations, *E*_*u*_ and *E*_*d*_, inhibited by a global inhibitory population, *I*, with weights ω_*iu*_ and ω_*id*_, respectively. A schematic is shown in **Figure 9A**. *S*_*e*_ and *S*_*i*_ are sigmoidal functions representing the steady state input-output relation of a neuron (on average), normalized between 0 and 1 (same as in the network model, Equation 3). *In*_*e*_ and *In*_*i*_ are afferent inputs to *E* and *I*, respectively.

(7) $$\{\begin{array}{ccc} \tau_{E}{\overset{•}{E}}_{u} & = & -E_{u}+S_{e}\left(\omega_{ee}E_{u}-\omega_{iu}I+In_{e}\right)\\ \tau_{E}{\overset{•}{E}}_{d} & = & -E_{d}+S_{e}\left(\omega_{ee}E_{d}-\omega_{id}I+In_{e}\right)\\ \tau_{I}\overset{•}{I} & = & -I+S_{i}\left(\omega_{ei}(E_{u}+E_{d})+In_{i}\right) \end{array}$$

A previous tone with higher frequency increases ω_*iu*_ while a tone with lower frequency increases ω_*id*_, the effect of which is similar to synaptic facilitation of inhibitory neurons in our full network model.

#### Phase plane analysis (Figures 9B,C)

Phase plane analysis is a technique to study the behavior of a dynamical system geometrically. For the 3-variable model, phase state space is projected onto the plane of *E*_*u*_ and *E*_*d*_ by setting *I* as instantaneous, meaning *I* = *S*_*i*_(ω_*ei*_(*E*_*u*_ + *E*_*d*_) + *In*_*i*_). The *E*_*u*_- nullcline is the curve where ${\overset{•}{E}}_{u}=0$, i.e., −*E*_*u*_ + *S*_*e*_(ω_*ee*_*E*_*u*_ − ω_*iu*_*I* + *In*_*e*_) = 0 and the *E*_*d*_- nullcline is the curve where ${\overset{•}{E}}_{d}=0$, i.e., −*E*_*d*_ + *S*_*e*_(ω_*ee*_*E*_*d*_ − ω_*id*_*I* + *In*_*e*_) = 0, where *I* = *S*_*i*_(ω_*ei*_(*E*_*u*_ + *E*_*d*_) + *In*_*i*_). The intersection of the *E*_*u*_- nullcline and *E*_*d*_- nullcline is the steady state solution of Equation (7), where *E*_*u*_, *E*_*d*_ and *I* do not change in time.

## Results

### Asymmetric inhibitory footprints give rise to direction selectivity

We formulate a distributed network model that consists of three subpopulations, each tonotopically organized: two excitatory populations (*E*_*up*_, *E*_*down*_) driving a common inhibitory population (*I*) that provides recurrent feedback to *E*_*up*_ and *E*_*down*_. The connectivity from the excitatory to the inhibitory neurons is symmetric, but the inhibitory feedback connection has an asymmetric projection profile (referred to as “footprint” below) (Figure 2, see Materials and Methods for details). Inhibitory neurons project only to the lower frequency side of *E*_*up*_ and to the higher frequency side of *E*_*down*_, thereby making the excitatory populations, *E*_*up*_ and *E*_*down*_ selective to ascending and descending frequency changes, respectively. The neurons of *E*_*up*_ and *E*_*down*_ have identical intrinsic properties. Recent experimental findings suggest that asymmetric inhibitory connectivity may underlie frequency change selectivity (Ye et al., 2010). Although, for simplicity, we consider strictly one-sided inhibitory footprints, similar selectivity effects would be found for two-sided footprints with an adequate amount of asymmetry (see Discussion). In the model, a response difference (*D*) is calculated as the time-average, relative difference in activity of *E*_*up*_ and *E*_*down*_ normalized by the sum of their activities during the current tone (Equation 6). A pitch change percept of ascending or descending is assigned according to whether *D* is positive or negative, respectively.

Neuronal units of *E*_*up*_, *E*_*down*_, and *I* receive feedforward input that is weighted by a Gaussian distribution based on the distance between a unit's characteristic frequency (CF) and the frequency of a tone component within the acoustic input. Excitatory coupling is local, with a width of 0.1 octaves, but inhibitory coupling is long range (length constant is 0.3 octaves). Due to the particular spectral property of Shepard tones (consisting of multiple octave-spaced pure tones), our model inherits a ring architecture with periodic boundary conditions. Therefore, we reduce the model's frequency range to one octave and represent each unit by the *pitch class* of its CF. For implementing dynamic simulations the one-octave PC range, a continuum, is discretized into 100 frequency values that are equally-spaced in logarithmic frequency scale. The model is an idealized mean-field model describing the dynamics of normalized firing rates of each unit, designed to account for the behavioral data on a phenomenological level.

**Figure 3:**
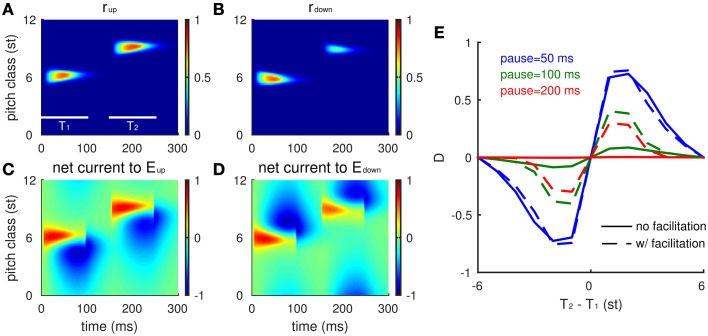
**Neuronal model responses for two successive Shepard tones mimic human perception**. **(A,B)** The spatiotemporal activity of the excitatory neurons (*E*_*up*_ in **A**, *E*_*down*_ in **B**) in response to a Shepard tone pair (T_1_ = 6 st, T_2_ = 9 st) is represented by their firing rates with the vertical axis corresponding to the PC of a unit's CF (see text). Each Shepard tone has a duration of 100 ms, with a 50 ms pause between tones. Firing rate is normalized between 0 and 1. **(C,D)** The synaptic input received by each neuron is shown for the *E*_*up*_ **(C)** and the *E*_*down*_ **(D)** populations. Although the early excitatory inputs are symmetric, the later inhibitory inputs are asymmetric, based on the asymmetric footprint from the inhibitory to excitatory units. **(E)** The response difference between *E*_*up*_ and *E*_*down*_ varies with PC interval between T_1_ and T_2_ consistently with human perception (Shepard, 1964; Chambers and Pressnitzer, 2014). The mean relative population activity differences *D* (Equation 6)during T_2_ are plotted as a function of the difference in pitch class between T_2_ and T_1_ (T_2_-T_1_). The response difference decreases with the pause between the tones [50 ms (blue), 100 ms (green), 200 ms (red)], decreasing steeper for static inhibitory synapses (solid) than for facilitating synapses (dashed).

We first consider the model's response to two Shepard tones (T_1_ and T_2_) without a pre-test sequence (Figure 3). Human listeners perceive relative steps of 1–5 semitones (st) as ascending, steps of 7–12 st (or equivalently -1 to -5 st) as descending, and a step of 6 st (tritone) as ambiguous (Shepard, 1964; Deutsch, 1986; Repp, 1997). Since the model is homogeneous along the frequency axis, we assume T_1_ = 6 st. At the onset of T_1_, both *E*_*up*_ and *E*_*down*_ have high firing rates (Figures 3A,B) with positive recurrent excitatory inputs centered around the network site for the PC of T_1_. This activity diminishes with time and its profile becomes asymmetric as inhibition develops (somewhat slower time scale) and suppresses lower frequency units in *E*_*up*_ and higher frequency units in *E*_*down*_ (Figures 3C,D). The post-stimulus (residual) inhibitory current decays with time constant 30 ms after the offset of T_1_. Hence, at the onset of T_2_ (PC = 9 st), *E*_*down*_ at the PC of T_2_ is inhibited while *E*_*up*_ is not, which gives *E*_*up*_ an advantage in competing with *E*_*down*_ for the model's prediction of pitch change percept. The positive difference (*D*) in response to T_2_ indicates an ascending percept, consistent with human perception for such a 3 st step change (Shepard, 1964; Chambers and Pressnitzer, 2014).

The model's responses are consistent with human psychophysics (Shepard, 1964; Chambers and Pressnitzer, 2014) for all possible step sizes [(−6, 6), Figure 3E]. The response difference (*D*) during T_2_ varies with different step sizes from T_2_ to T_1_: *E*_*up*_ responds stronger to a T_2_ that is within +6 st step from T_1_, while *E*_*down*_ responds stronger to a T_2_ that is within -6 st step from T_1_. The magnitude of the response difference is maximal at 1–2 st from T_1_ and decreases with greater distance between T_1_ and T_2_ due to the decrease of inhibitory strength with distance (see Equation 5). *E*_*up*_ and *E*_*down*_ reach the same activity level for a tritone step (6 st, same as PC = −6 st due to periodicity), since they are equally separated from above and below.

Since inhibition decays during the pause between T_1_ and T_2_, the response difference (*D*) decreases with pause time (Figure 3E, different colors). For pauses greater than 100 ms, the pitch change sensitivity has practically disappeared. In human perception, comparisons can be performed above the 50% level for considerably longer pauses between tones in the pair. Our model can account for such performance over longer pauses by extending temporally the effects of inhibition, thereby enhancing the difference (*D*) at longer times. Below (see Section The Tritone Comparison is Biased by One-sided Preceding Tones), we incorporate slow facilitation of inhibitory synapses to implement the enhancement; as a preview notice the dashed curves in Figure 3E.

### Single unit responses contain spectral information of both current tone and previous tone

**Figure 4:**
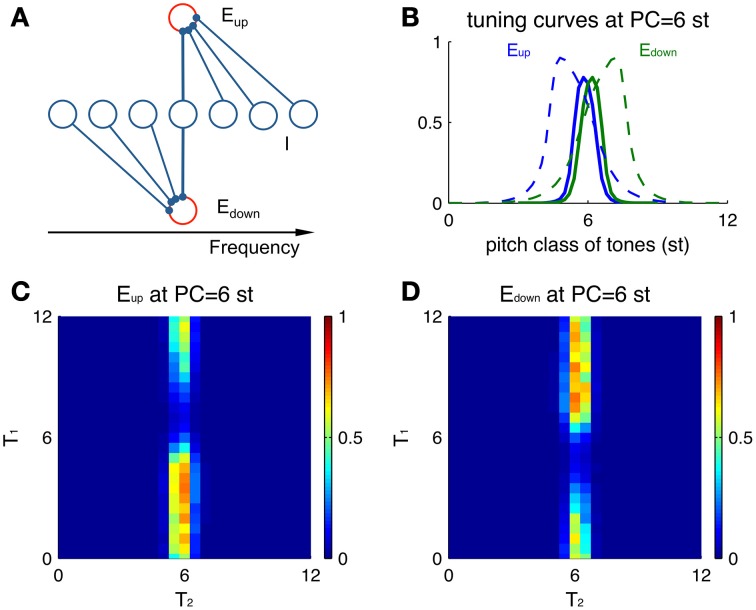
**Single-unit properties of *E*_*up*_ and *E*_*down*_**. **(A)** Schematic showing the different sources of inhibitory input to *E*_*up*_ and *E*_*down*_ units. **(B)** Tuning curves of *E*_*up*_ (blue solid) and *E*_*down*_ (green solid) units (at PC = 6 st) are skewed in different directions. Larger skewness is seen when the tuning curves (dashed) are calculated for a different parameter set with broader input. The input drive for a tone is modeled as a sustained Gaussian function centered at the pitch class of that tone (Equation 1). The tuning curve shows peak amplitude of firing rate during the stimulus duration (100 ms). **(C)** A preceding tone influences the neural activity to the next tone via asymmetric inhibition. Color represents the peak amplitude of firing rate of an *E*_*up*_ unit (PC = 6 st) during T_2_ for different combinations of sequential stimuli T_1_ and T_2_. A Shepard tone of random pitch class is presented before T_1_ for random initial conditions and plotted results are averaged over 10 runs. **(D)** Plot as in (**C)** for an *E*_*down*_ unit at the same location (PC = 6 st).

The direction-selective excitatory neurons exhibit non-symmetric tuning curves, even without a preceding stimulus (Figure 4). A tuning curve in the present context describes the response properties of a neuron to Shepard tones of any PC. Since an *E*_*up*_ unit receives inhibition from the higher frequency side (Figure 4A), tones above the unit's PC invoke more inhibition on this *E*_*up*_ unit, resulting in lower firing rates than tones at lower PC. Conversely, an *E*_*down*_ unit is inhibited from the lower frequency side, thus responding stronger to tones above its PC. Hence, the tuning curve of *E*_*up*_ units leans to lower PC's (positive skewness, Figure 4B blue) and the opposite for *E*_*down*_ units (negative skewness, Figure 4B green). In this example, both units receive the same input with Gaussian weight centered at 6 st (see Materials and Methods, Equation 1).

Tuning curves for *E*_*up*_ and *E*_*down*_ units also depend differentially on the previous tone. We measure responses to the second tone T_2_ of a Shepard tone pair for different combinations of T_1_ and T_2_ (Figures 4C,D). Overall, the activities are restricted to pairs with T_2_ around the PC of both *E*_*up*_ and *E*_*down*_ units (here 6 st), since their afferent inputs are localized around their PC. A preceding Shepard tone T_1_ above 6 st elicits a reduction in the response of the *E*_*up*_ unit (Figure 4C) while the *E*_*down*_ unit (Figure 4D) is not affected. Conversely, a T_1_ below 6 st suppresses the response of the *E*_*down*_ unit only. Therefore, the response of a single unit reflects the spectral information of the current tone (T_2_) due to narrow tuning and the relative position of a previous tone (T_1_) due to direction selectivity.

### The tritone comparison is biased by one-sided preceding tones

Psychophysical experiments show that using a preceding sequence of Shepard tones with PC's between a tritone pair (T_1_ and T_2_) biases the pitch change perception: if the preceding tones are spectrally located above (i.e., within +6 st from) T_1_, then T_2_ is more likely perceived as an ascending step from T_1_. If the preceding tones are within −6 st from T_2_, a descending step is more likely perceived (Repp, 1997; Englitz et al., 2013) (Figure 1). The silent gap between the context sequence and the tritone pair in the psychophysical experiments typically exceeds 0.5 s. This gap is much longer than the time scales of our model's excitatory and inhibitory populations (less than 30 ms). Therefore, a slow adaptation mechanism is needed to hold the effects of context−a mechanism that can imbalance the delayed competition between *E*_*up*_ and *E*_*down*_ during the test in favor of one or the other depending on the relative position of the context tones and the tritone pair. For this adaptation, our model implements slow facilitation of synaptic inhibition; other candidate mechanisms for adaptation are considered in the Discussion.

**Figure 5:**
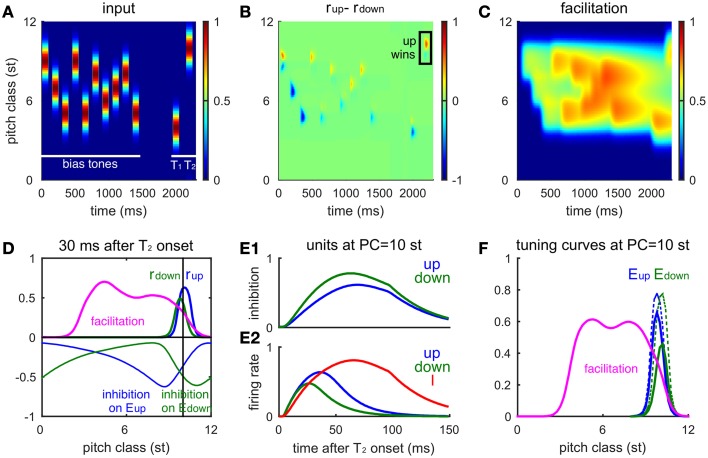
**The network model accounts for the influence of the biasing sequence on tritone perception**. **(A)** A randomly drawn sequence of 10 Shepard tones precedes an ambiguous pair (at 4 and 10 st). This bias sequence is restricted to lie between the ambiguous pair. Tone durations are 100 ms and inter-tone pause is 50 ms. The gap between the biasing sequence and the tritone pair is 0.5 s. **(B)** The firing rate difference of *E*_*up*_ and *E*_*down*_ populations (*r*_*up*_(*x, t*)-*r*_*down*_(*x, t*), see Materials and Methods) for the entire sequence shows the local response to each tone. *E*_*up*_ has a larger response to the final tone, T_2_, indicating an ascending percept (box, consistent with human perception). **(C)** The influence of the bias sequence is reflected in the accumulation of the facilitation level *F* in the biased region. **(D)** Snapshot of the network activity at 30 ms after the onset of T_2_ (PC = 10 st). Facilitation level (magenta) has built up in the biasing region, below the pitch class of T_2_. The firing rate profile for *E*_*up*_ (blue thick) has a higher peak than for *E*_*down*_ (green thick) showing that *E*_*up*_ is winning the competition for the model's perceptual choice. Inhibitory input to the *E*_*up*_ (blue thin) and the *E*_*down*_ (green thin) units spread to the higher frequency side and the lower frequency side, respectively. The *E*_*down*_ unit receives higher inhibition than the *E*_*up*_ unit at PC = 10 st (black vertical line) due to facilitation of the *I* units below T_2_. **(E)** Time courses of the *E*_*up*_ (blue) and *E*_*down*_ (green) units at the pitch class of T_2_ during T_2_ presentation. (**E1**), Inhibitory inputs to the *E*_*up*_ and *E*_*down*_ units; **(E2)**, firing rates of the *E*_*up*_, *E*_*down*_, and *I* (red) units. **(F)** Tuning curves of *E*_*up*_ and *E*_*down*_ units (at PC = 10 st) are affected differentially by biasing. The tuning curve of the *E*_*down*_ (solid green) unit reduces more than the *E*_*up*_ (solid blue) unit after biasing from below. The tuning curves of *E*_*up*_ (dashed blue) and *E*_*down*_ (dashed green) units without biasing are the same as the solid curves in Figure 4B. The biasing sequence is the same as in **(A)**; the tuning curves are measured after the biasing sequence and the gap (0.5 s).

Slow facilitation of inhibitory synapses integrates spectral information of stimulus history in the model. This slow adaptation thereby biases the model's pitch-change-direction percept of the tritone pair that would be ambiguous if tested alone. During a preceding sequence of Shepard tones, *E*_*up*_ and *E*_*down*_ respond to each tone locally with different activity levels indicating percepts of pitch-change direction. Inhibitory synapses gradually facilitate wherever inhibitory neurons are activated (Equation 2), representing a spectral distribution of recent stimulus history (Figure 5C). The facilitation level decays slowly during the silent gap between the preceding sequence and the tritone pair. The facilitated inhibitory synapses disadvantage *E*_*down*_ during the T_2_ presentation after a sequence of Shepard tones below T_2_, resulting in a larger population response difference (box in Figure 5B, red area larger than blue area). This imbalance leads to an ascending percept in the model for the tritone comparison. Population firing rates of *E*_*up*_ (Figure 5D, thick blue) and *E*_*down*_ (Figure 5D, thick green) start to separate at 30 ms after the onset of T_2_. Inhibitory current on *E*_*up*_ (Figure 5D, thin blue) comes from the higher frequency side and spreads to the lower side, pushing the population peak of *E*_*up*_ above the PC of T_2_. *E*_*up*_ continues recruiting more units at higher CF's by recurrent excitation while *E*_*down*_ is suppressed due to the facilitated inhibition from lower CF units. Hence, the model predicts an ascending percept for a tritone pair after a preceding sequence of tones within +6 st from T_1_. This context dependence of the model is consistent with psychophysical results (Repp, 1997; Englitz et al., 2013).

The differential effects of facilitation on *E*_*up*_ and *E*_*down*_ are due to their different sources of inhibition. It is sufficient to consider the units at the PC of T_2_ during the T_2_ presentation, since *E*_*up*_ and *E*_*down*_ respond locally to each tone. The *E*_*up*_ unit receives inhibition from above while the *E*_*down*_ unit receives inhibition from below (Figure 4A), where inhibitory synapses have been facilitated during the context tones (Figure 5D, magenta). With a stronger synaptic weight, inhibition on the *E*_*down*_ unit rises faster than that on the *E*_*up*_ unit from the onset of T_2_ (Figure 5E1), resulting in a lower and earlier peak in firing rate of the *E*_*down*_ unit (Figure 5E2). Excited by both *E*_*up*_ and *E*_*down*_, the *I* unit rises with *E*_*up*_ after *E*_*down*_ turns to decrease, which further suppresses *E*_*down*_. Therefore, facilitation on one side of the inhibitory units increases inhibition on either *E*_*up*_ or *E*_*down*_, which in turn biases the competition toward the other population.

Tuning curves of the *E*_*up*_ and *E*_*down*_ units change differently after being biased on one side. After biasing from below, inhibition from *I* units in that region is facilitated (Figures 5C,F, magenta). Therefore, the overall response level of the *E*_*down*_ unit (Figure 5F, solid blue) is lower than that of the *E*_*up*_ unit (Figure 5F, solid green) and both show a reduction of activity compared to that without biasing (Figure 5F, dashed lines). Such a difference in tuning curves of *E*_*up*_ and *E*_*down*_ persists on the time scale of facilitation (τ_*fd*_ = 2s) and is still significant after a half second of silence.

Let's reconsider the situation of comparing two successive Shepard tones without preceding context. Facilitation enables such a comparison over a long pause by viewing T_1_ as a context tone for T_2_ (Figure 3E, dashed). For a T_2_ within +6 st from T_1_, facilitation level builds up around the PC of T_1_, which is below T_2_. The *E*_*down*_ units around the PC of T_2_, therefore, receive more inhibition than *E*_*up*_ units. The competition between *E*_*up*_ and *E*_*down*_ during T_2_ is thus favored toward *E*_*up*_, which gives a positive response difference (*D*). Conversely, a T_2_ within -6 st from T_1_ has a negative response difference.

### Biasing effects depend on the spectral distribution of bias tones and tuning width of i units

#### Frequency dependence of single-tone biasing

**Figure 6:**
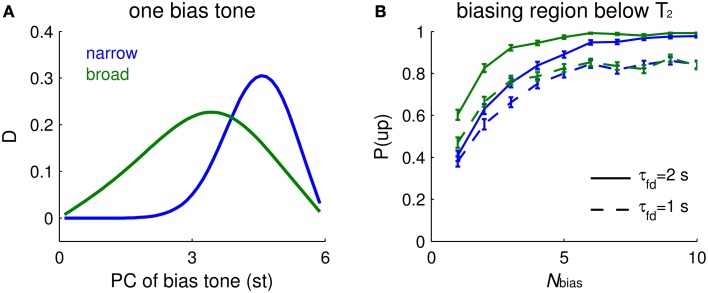
**Biasing effects depend on the spectral distribution of bias tones and tuning width of *I* units**. **(A)** Mean relative response difference, *D* (Equation 6, see Materials and Methods), of *E*_*up*_ and *E*_*down*_ for T_2_ vs. PC of a single bias tone (abscissa, different locations) depends on the tuning width of the inhibitory units (narrow tuning = blue, broad tuning = green). The ambiguous Shepard tone pair is for T_1_ = 0 st, T_2_ = 6 st. The footprints of *E* to *E* (σ_*ee*_) and *E* to *I* (σ_*ei*_) are 2.5 times wider for broad tuning of *I* units, and the synaptic strength of recurrent excitation (*a*_*ee*_) is increased to have comparable firing rates. Parameter values for narrow tuning are σ_*ee*_ = 0.02, σ_*ei*_ = 0.08 octaves, and *a*_*ee*_ = 0.7, and those for broad tuning are σ_*ee*_ = 0.05, σ_*ei*_ = 0.2 octaves, and *a*_*ee*_ = 1.5. Other parameters are the same as used in Materials and Methods. Narrow tuning is used in other figures. **(B)** The biasing effect accumulates with the number of bias tones. The buildup depends more steeply on *N*_*bias*_ for broad tuning of *I* units (green) than for narrow tuning (blue). A faster decay time constant of facilitation τ_*fd*_ leads to lower biasing effects, but does not strongly affect the buildup “rate” (solid: τ_*fd*_ = 2 s; dashed: τ_*fd*_ = 1 s). The percentage of ascending responses, *P*(*up*), over trials (each trial is for a sequence of random Shepard tones) is plotted vs. the number of biasing tones *N*_*bias*_. An “ascending choice” is made if *D* > 0.1; a threshold value, 0.1, is used for all conditions. The *N*_*bias*_ Shepard tones for a sequence are randomly sampled for ascending bias in the region above T_1_ and below T_2_ and for the tritone pair as in **(A)**; there were 400 trials for each *N*_*bias*_ (error bars denote 2 SEM).

With a single Shepard tone as context that precedes a tritone pair, the impact of biasing depends on the PC of the bias tone, B, and on the tuning width of *I* units. If the tuning width is narrow (about 3 st for our default parameter settings, not shown explicitly), biasing is most effective when it occurs about 1 st from T_2_ (Figure 6A, blue). If the tuning of an *I* unit is broad (say, about 6 st), the most effective bias tone is shifted to midway between T_1_ and T_2_ (Figure 6A, green). The response difference of *E*_*up*_ and *E*_*down*_ depends on the facilitation level difference from above and below T_2_. On the one hand, B needs to be close enough to T_2_ so that the *I* units activated by B partially overlap those activated by T_2_; the biasing effect depends on accumulated facilitation level, more on one side than the other, so that inhibition affects *E*_*up*_ and *E*_*down*_ units differentially. On the other hand, when B is too close to T_2_, the facilitation level is maximal but flat around the PC of T_2_, showing little difference between the two sides of T_2_. Therefore, the dependence of the tritone comparison on the PC of B scales with the tuning widths of inhibitory units.

#### Biasing effects accumulate with the number of bias tones

The buildup function for the strength of the biasing effect depends on the frequency dependence function of a single-tone bias, in addition to the decay time constant of facilitation. The effectiveness of biasing increases with the total number of biasing tones, *N*_*bias*_. The model's ascending choice probability gradually increases and approaches the asymptotic value with different buildup rates depending on the frequency dependence function of a single-tone bias: a broader dependence function results in faster buildup (Figure 6B, green) than a narrower dependence function (Figure 6B, blue). The psychometric buildup function measured by Chambers and Pressnitzer (2011) starts at 0.75 when *N*_*bias*_ = 1 and reaches a plateau when *N*_*bias*_ is around 5. Hence, the buildup function with a broader inhibitory tuning is closer quantitatively to the psychometric buildup function.

Surprisingly, the buildup rate of the model's neurometric function changes little when the decay time constant of facilitation, τ_*fd*_, is accelerated by a factor of 2 (Figure 6B, blue dashed). This time constant affects more the absolute value rather than the “spatial” distribution of facilitation, thus reducing the plateau value instead of the buildup rate. The spatial gradient of facilitation around the PC of T_2_ determines the decision variable, *D*, on which the perceptual choice is based. Due to the randomly drawn PC-values of the bias tones, it is possible that for low *N*_*bias*_, the majority of trials have bias tones distant from T_2_. We expect that biasing is weaker (Figure 6A, for *N*_*bias*_ = 1) for distant bias tones when, as here, *I* units are narrowly tuned. With more bias tones in a trial the biasing region becomes more uniformly covered. When *I* units are broadly tuned, the biasing effects function is also broader for single-tone bias (Figure 6A, green), resulting in a faster buildup rate (Figure 6B, green). Therefore, the shape of the neurometric function of *N*_*bias*_ depends mainly on the frequency dependence function of single-tone biasing effects, in addition to the decay time constant of facilitation.

### Non-uniform inhibitory synaptic strengths can account for individual variations in tritone comparisons

**Figure 7:**
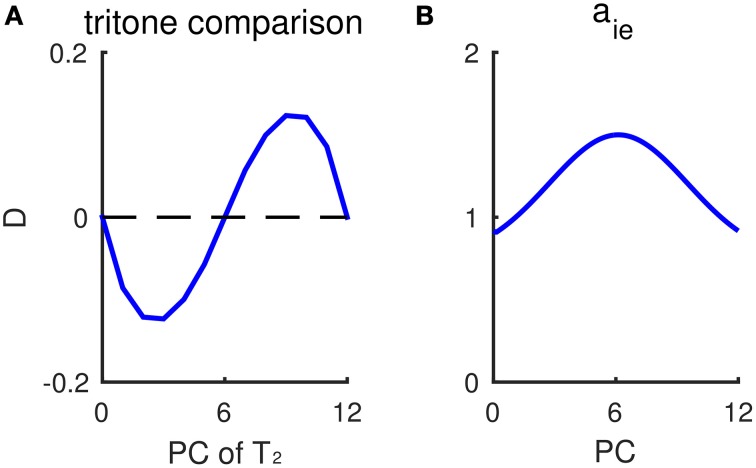
**Non-uniform inhibitory synaptic strengths lead to a sinusoidal-like pattern of outcomes for tritone comparisons**. **(A)** Response difference of *E*_*up*_ and *E*_*down*_ to tritone pairs at different pitch classes without context. The inhibitory pre-synaptic strength *a*_*ie*_ depends on the pitch class of *I* neurons. The profile of *a*_*ie*_ is shown in (**B)**. Mean relative population activity difference, *D* (Equation 6, see Materials and Methods), of *E*_*up*_ and *E*_*down*_ during T_2_ has a sinusoidal-like pattern, varying with the pitch class of the second tone T_2_. A positive *D* predicts “ascending” response and negative *D* predicts “descending.” The pitch classes of T_2_ with largest response difference |*D*| correspond to where *a*_*ie*_ changes most steeply. **(B)** The dependence of inhibitory pre-synaptic strength, *a*_*ie*_, on pitch class of *I* neurons. In this simulation, the inhibitory synaptic current, $h_{ie}^{\alpha }$, in Equation (4) is given as: $h_{ie}^{\alpha }(x,t)=\int \omega_{\alpha }(y)a_{ie}(x-y)(1+\gamma_{f}F(x-y,t))r_{I}(x-y,t)dy$, α= up, down.

Our model provides a plausible explanation for individual variations in the tritone comparison among and across individuals. The variability across subjects, i.e., perceiving different directions on average for the same tritone pair, has been termed the tritone paradox (Deutsch, 1986; Deutsch et al., 1990). Moreover, individual responses to tritone pairs (half-octave apart) often show a dependence on PC with a sinusoidal-like pattern (Figure 7A). Instead of being around chance level for a tritone pair of any PC, some pitch classes are more likely to be heard as the higher of a tritone pair, while some pitch classes are more likely to be heard as the lower (Deutsch et al., 1990, see Figure 3; Deutsch, 1991, see Figure 3). Such sinusoidal patterns for tritone comparison vary among subjects and are found to correlate with language (Deutsch, 1991) and the vocal range of one's speech (Deutsch et al., 1990). Our model can reproduce the sinusoidal-like pattern of individual tritone responses using a heterogeneous inhibitory population with pre-synaptic strength, *a*_*ie*_, depending on PC (Figure 7B). Different distributions of inhibitory synaptic strengths give different sinusoidal-like patterns as a function of PC, which can account for the individual variations across subjects.

According to the model, the pitch class that would be most frequently perceived as ascending (with largest *D*) corresponds to the PC at which inhibitory synaptic strength decreases most steeply. Therefore, inhibitory synaptic strengths, which may be shaped by prior auditory experience, can be an intrinsic bias that varies among subjects for the ambiguous tritone comparison. When the distribution of inhibitory synaptic strengths (*a*_*ie*_) is Gaussian-shaped with a peak at PC = 6 st (Figure 7B), for example, the response difference (*D*) for a tritone comparison is of largest magnitude when T_2_ is around 3 and 9 st (Figure 7A), where *a*_*ie*_ decreases most steeply. Therefore, the sinusoidal-like pattern of a tritone response depends on the distribution of inhibitory synaptic strengths. By shifting the profile of *a*_*ie*_, we can generate sinusoidal-like patterns with the largest *D* at different PC, corresponding to different tritone comparison patterns among subjects. Deutsch et al. (1990) have shown that the pitch classes perceived as mostly likely ascending are typically at the band limit of the listener's vocal range of fundamental frequencies. Hence, our model implies a correlation of inhibitory synaptic strength and vocal occurrence of one's speech.

### Frequency shift detection for spectrally non-periodic stimuli

The periodic structure of a Shepard tone is not essential for the model to detect frequency change. The model can be readily generalized to compare spectrally non-periodic complex tones, in which case the network model would be distributed on an extended tonotopic axis without periodic boundary conditions. The model's response to each frequency component within T_2_ depends on its distance from the frequency components in T_1_ that are just above or below it. Therefore, the model makes a local comparison of frequency components within consecutive tones. Population activities of *E*_*up*_ and *E*_*down*_ across the tonotopic axis are compared to make decisions of frequency change direction.

**Figure 8:**
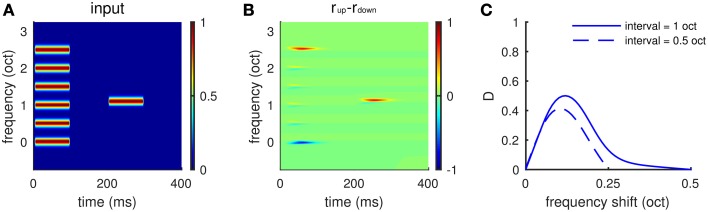
**Frequency shift detection for spectrally non-periodic stimuli**. **(A)** An example of input stimuli. A chord of six synchronous pure tones equally spaced along the logarithmic frequency scale is followed by a test pure tone. The interval between adjacent components in the chord is 0.5 octaves. The ordinate is frequency relative to the lowest component of the chord. The second tone is 0.1 octaves higher than the third lowest component in the chord. **(B)** *E*_*up*_ shows larger response than *E*_*down*_ to the second tone, indicating a perceived upward shift of frequency. **(C)** Mean relative response difference,*(D)* (Equation 6, see Materials and Methods), is largest when the frequency shift is about 0.1 octaves for both intervals, 0.5 octaves (dashed), and 1.0 octaves (solid). Results are averaged for frequency shift relative to “inner” components (2–5) of the chord. There is little variation in the profile in **(C)** for different inner components. The shape of the tuning curve for frequency shift is qualitatively the same as that measured in psychophysical experiments (Demany et al., 2009, Figures 1C,D).

The local comparison property of the model provides a neuronal-based explanation for the experiments by (Demany and Ramos, 2005; Demany et al., 2009). Each sound stimulus was a chord of six synchronously played pure tones, whose frequencies were equally spaced on a logarithmic scale, followed by a test pure tone (Figure 8A). Subjects were asked to compare the test pure tone with the chord in pitch height without knowing which component of the chord should be the basis for their comparison. They found that subjects were most sensitive to a one semitone change in frequency between the test pure tone and one of the chord components (Demany et al., 2009, see Figure 1). Our model can be considered a neuromechanistic implementation of their hypothesis of frequency shift detectors. The model gives larger firing rates of *E*_*up*_, for example, when the test tone is 0.1 octaves above the third lowest frequency component of the chord (Figures 8A,B), predicting an ascending percept. The dependence of response difference (*D*) on frequency shift (Figure 8C) resembles the psychometric tuning curves of frequency shift detectors measured by Demany et al. (2009) (see Figure 1). Our model shows maximum response difference (*D*), corresponding to the highest sensitivity of human subjects, for a frequency shift of about 0.1 octaves for two different spectral intervals (0.5 and 1.0 octaves) separating components of the chord (Figure 8C).

### 3-variable winner-take-all (WTA) model captures biasing behavior

The behavior of biased competition can be understood by considering a simple winner-take-all (WTA) model. Consider a general model of two excitatory populations *E*_*u*_ and *E*_*d*_ inhibited by a global inhibitory population *I* with weights ω_*iu*_ and ω_*id*_, respectively. The weights are activity dependent, affected differentially by previous tones: higher frequency tones increase ω_*iu*_ while lower frequency tones increase ω_*id*_, similar to the facilitation dynamics of inhibition in the full model.

**Figure 9:**
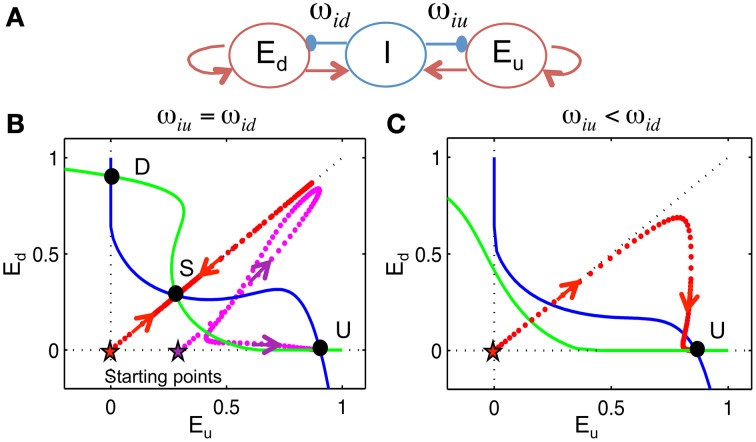
**3-variable winner-take-all model**. We devised a 3-variable model, without frequency dependence, to analyze the biasing mechanism of the competition between *E*_*up*_ and *E*_*down*_ populations. **(A)** The model, represented by this schematic, consists of two excitatory populations, with firing rates *E*_*u*_ and *E*_*d*_, that are inhibited by a global inhibitory population *I* with weights ω_*iu*_ and ω_*id*_, respectively (see Materials and Methods). Inhibition is without dynamic facilitation. **(B,C)** Phase plane analysis (see Materials and Methods). We project the phase space onto the plane of *E*_*u*_ and *E*_*d*_. Null-clines (where rate of change is zero) of *E*_*u*_ (blue) and *E*_*d*_ (green) are calculated by assuming *I* acts instantaneously. **(B)** When ω_*iu*_ = ω_*id*_, there are three steady states (U, D, S). Trajectory (dotted) converges to the U state if *E*_*u*_ is larger than *E*_*d*_ initially [magenta, initial condition (*E*_*u*_(0), *E*_*d*_(0), *I*(0)) = (0.3, 0, 0)] and approaches the S state if *E*_*u*_ and *E*_*d*_ are equal, initially [red, (*E*_*u*_(0), *E*_*d*_(0), *I*(0)) = (0, 0, 0)]. **(C)** When ω_*iu*_ < ω_*id*_, there is only one steady state. The trajectory converges to the U state even if *E*_*u*_ equals *E*_*d*_ initially [red, (*E*_*u*_(0), *E*_*d*_(0), *I*(0)) = (0, 0, 0)].

By assuming rapid recruitment of *I* units (*I*-activity, an instantaneous function of inputs) we can project the state space onto the phase plane of *E*_*u*_ and *E*_*d*_. When ω_*iu*_ = ω_*id*_, there are three steady states: the U state (*up*-dominant) where *E*_*u*_ > *E*_*d*_, the D state (*down*-dominant) where *E*_*u*_ < *E*_*d*_ and the S state (symmetric) where *E*_*u*_ = *E*_*d*_. The U and D states are stable, while the S state is a saddle point. This is the phase plane of competition dynamics. If *E*_*u*_ and *E*_*d*_ start off as identical, the solution trajectory is symmetric and converges to the S state if there are no fluctuations (Figure 9B, red), while the U state is approached if *E*_*u*_ is higher, initially (Figure 9B, magenta). On the other hand, suppose that ω_*iu*_ < ω_*id*_, as would occur if ω_*id*_ were facilitated by preceding lower frequency tones. In this case, the competition is biased toward *E*_*u*_ such that only the U state remains and the solution converges to the U state for any initial condition (Figure 9C, red). This shows that initial conditions and inhibitory synaptic strengths can both bias the competition between *E*_*u*_ and *E*_*d*_.

Similarly, in the full model there are also two ways to bias the competition between *E*_*up*_ and *E*_*down*_ units. One way is based (locally in time) on the residual inhibition from a previous tone, which is long-range along the tonotopy but short-lived. This residual inhibition determines the network's initial state for the next tone, so that the population is slightly inhibited by the previous tone and thus has a much lower response to the next tone. A second way is based on the facilitation level that reflects the distribution of previous tones and biases the competition according to relative positions. Synaptic strengths of inhibitory units that are above the PC of T_2_ correspond to ω_*iu*_ in the 3-variable model and synaptic strengths of inhibitory units that are below the PC of T_2_ correspond to ω_*id*_, since *E*_*up*_ and *E*_*down*_ are inhibited from opposite sides. Different from the residual inhibition that resulted from the most recent tone, facilitation is a slow process and contains information of multiple previous tones. However, facilitated synaptic strengths can only play a role when they are activated during the test tone presentation.

## Discussion

We have developed a neuromechanistic model for comparing the pitch of successive tones and to account for the effects of preceding tone context. Spectral comparisons of this kind are common in everyday communication as well as in music. The central elements of the model are excitatory populations whose activity is sensitive to the direction of frequency-change due to asymmetric inhibitory input. The model successfully accounts for a set of psychoacoustic studies (Repp, 1997; Chambers and Pressnitzer, 2011; Englitz et al., 2013) investigating contextual influences on the directional percept of otherwise ambiguous steps in pitch between a half-octave separated Shepard tone pair. Slowly accumulating over past stimuli, facilitation of inhibitory synapses disrupts the balance of competition between the two direction-selective populations, thus biasing the pitch change percept. The model predicts that the most effective bias tone depends on the tuning width of the inhibitory population and exhibits buildup of biasing effects with increasing number of context tones. Finally, the model when extended over the whole tonotopic axis shows similar tuning curves of frequency shift for spectrally non-periodic tones as measured in psychophysical experiments (Demany et al., 2009).

### Physiological correlates of the model

Asymmetric inhibition in the frequency response fields of neurons in auditory cortex has been suggested to be one of the underlying mechanisms for direction selectivity (Suga, 1965; Shamma et al., 1993; Fuzessery and Hall, 1996; Zhang et al., 2003). Frequency response areas show strong correlation between asymmetric inhibitory sidebands and the direction-selectivity of neurons (Shamma et al., 1993). Moreover, the spectral offset of excitatory and inhibitory synaptic receptive fields are shown to contribute to frequency sweep direction selectivity (Zhang et al., 2003; Ye et al., 2010; Kuo and Wu, 2012). Such asymmetries are in line with the asymmetric inhibitory footprints in our model. However, the sweep rates in these studies (on the order of 10 octaves per second) are much faster than our model could distinguish in its current form. The neuronal time scales required for such fast sweep detection may exceed the biophysical capabilities in auditory cortex; such neuronal computations better match the properties of auditory brain stem. Reducing model time constants (say by a factor of at least 10) may allow for the detection of fast frequency sweeps.

Beyond the architecture another feature of our model is facilitation of the inhibitory population's synaptic output. A possible candidate for the inhibitory population in our model is the low-threshold spiking (LTS) interneurons, which exhibit short-term synaptic facilitation (Beierlein et al., 2003). It is conceivable that facilitated recruitment of inhibition by excitatory neurons (Reyes, 2011) might also support context dependence. Such a formulation would require additional variables and be less parsimonious. It has also been found that hearing experience induces a shift of synaptic inhibitory short-term plasticity from depression to facilitation, mainly due to the development of LTS cells (Takesian et al., 2010).

### The asymmetric I-E connectivity

Our model uses a common inhibitory population that projects to *E*_*up*_ and *E*_*down*_ populations in opposite frequency directions along the tonotopic axis. The asymmetry in inhibitory footprints not only generates direction selectivity for successive tones, but also exerts different suppression on *E*_*up*_ and *E*_*down*_ from the *I* units facilitated by context tones depending on their relative spectral positions. The common inhibition enables competition between *E*_*up*_ and *E*_*down*_ populations, thus enlarging the response difference between them and making decisions more robust. Our network architecture differs from that in the model of Husain et al. (2004) where two separate *E*-*I* pairs are used as *up*- and *down*-selective units without an adaptation mechanism. Furthermore, their model uses asymmetric *E* to *I* connections, which implies that inhibition level depends on the activities of excitatory populations. Therefore, their model would predict a correlation between the current pitch change decision and the previous. Physiological measurements of inhibitory neurons could be used to distinguish between the two models.

**Figure 10:**
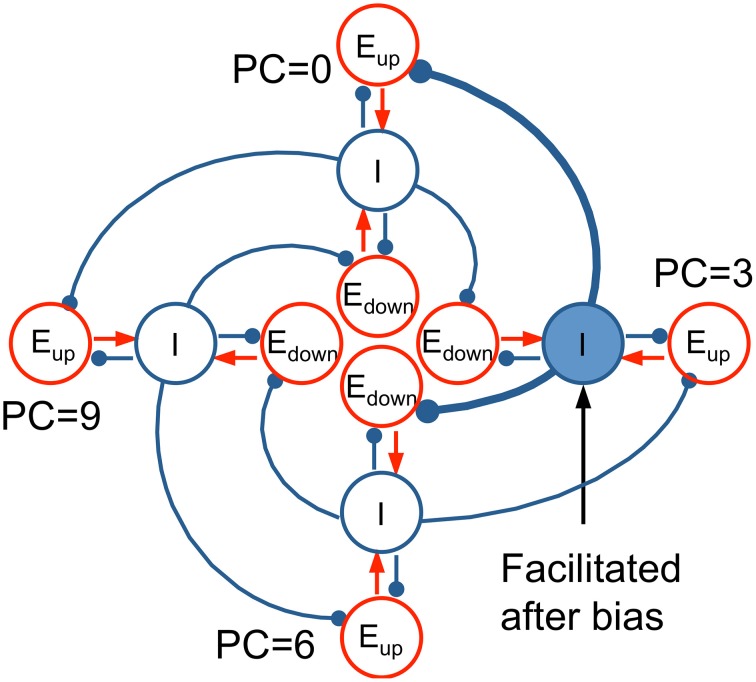
**Idealized conceptual model for *E*_*up*_ and *E*_*down*_ units on a pitch class circle**. Four tri-unit subpopulations (*E*_*up*_, *E*_*down*_, *I*) at representative PC's (0, 3, 6, 9 st), including their interactions, are shown to illustrate the mechanism of the full network model (Equation 2). *I* units (blue) inhibit the *E*_*up*_ unit below (lower CF) and the *E*_*down*_ unit above (higher CF). When a bias tone is presented at PC = 3, the synaptic strength of the *I* unit at PC = 3 is facilitated, resulting in more inhibition to the *E*_*up*_ unit at PC = 0 and the *E*_*down*_ unit at PC = 6. Hence, T_1_ at PC = 0 invokes a weaker response in *E*_*up*_ (*D* < 0 for T_1_, perceived as descending), while T_2_ at PC = 6 results in a weaker response in *E*_*down*_ (*D* > 0 for T_2_, perceived as ascending).

The essential mechanism of how our model's architecture leads to context effects can be illustrated with a conceptual model, an idealization based on our computational network model. The conceptual model consists of four tri-unit subpopulations (*E*_*up*_, *E*_*down*_, *I*) at representative PC's (0, 3, 6, 9 st) distributed around the PC circle (Figure 10). In the model, each *I* unit inhibits the *E*_*up*_ unit below (lower frequency) and the *E*_*down*_ unit above (higher frequency). When a context tone is presented at PC = 3 st, for example, the *I* unit at PC = 3 st is facilitated, which increases inhibition on the *E*_*up*_ unit at PC = 0 st and the *E*_*down*_ unit at PC = 6 st. Therefore, the pitch change percept is biased toward descending to T_1_ at PC = 0 st and ascending to T_2_ at PC = 6 st.

The connectivity between inhibitory and excitatory populations in our model does not need to be restricted to one-side only; instead a distributed degree of asymmetry of inhibitory footprints can be incorporated. We can categorize excitatory units into *E*_*up*_ or *E*_*down*_ populations based on their relative footprint widths from inhibitory neurons in the opposing tonotopic directions; those with symmetric inhibition would be pitch detectors (non-direction-selective). Since different inhibition levels on *E*_*up*_ and *E*_*down*_ would result from their different connections from *I* units, we expect that adding non-selective neurons would not alter the biasing effects on the direction-selective populations. In future work, we will extend the model to include both direction-selective and non-selective populations and investigate their coexistence and interactions.

### Other adaptation mechanisms

Context dependence here refers to the effect of preceding stimuli on the response to a discrimination task or specified stimulus. Adaptation (typically, reduction) of neuronal activity from previous inputs can affect current responsiveness and is often proposed as causal for contextual effects. Potential neuronal mechanisms may involve fatigue of repetitive spike generation or depression of excitatory synapses, slowly accumulating negative feedback. Context dependence has been reported as stimulus specific adaptation for stations along the auditory pathway in the oddball paradigm (Ulanovsky et al., 2003, 2004; Antunes et al., 2010; Lumani and Zhang, 2010). Models that incorporate synaptic depression can account for several features of such stimulus specific adaptation with depression implemented in recurrent connections (Nelken, 2014) or in feed forward synaptic dynamics (Mill et al., 2011, 2012; Taaseh et al., 2011). Spike frequency adaptation has also been reported as contributing to context dependence in auditory (Abolafia et al., 2011) and somatosensory cortex (Davies et al., 2012). Change detection has been linked to both mechanisms (Puccini et al., 2006). Pitch change can also be detected as a mismatch of the expected and the predicted pitch (Balaguer-Ballester et al., 2009).

In contrast, our model implements slow facilitation of inhibition as an adaptive mechanism for the context-dependence of frequency change direction. In developing our model we considered other mechanisms: spike-frequency adaptation and synaptic depression. Suppose, the *E*_*up*_ and *E*_*down*_ units “fatigue” slowly with spike-frequency adaptation when activated. In the region for ascending bias (above T_1_ and below T_2_), the biasing tones are more likely to elicit local wins by *E*_*up*_ units near the PC of T_2_ and the *E*_*down*_ units near the PC of T_1_. Thus, *E*_*up*_ units near the PC of T_2_ would have fired more and be more adapted, and hence would favor a descending response, contradictory to the psychophysical results. Spike-frequency adaptation alone seems inadequate to explain the biasing phenomenon observed in Shepard tones. Alternatively, suppose that synaptic depression on recurrent excitation (*E* to *E*) depends on the activities of *E*_*up*_ and *E*_*down*_. Similar to spike-frequency adaptation, recurrent depression predicts a correlation of *E*_*up*_ and *E*_*down*_ activities with their previous activities, respectively. In other words, it predicts a correlation of present *up/down* percept with previous *up/down* percepts. However, psychophysical experiments have found little dependence of the response on the *up*'s and *down*'s during the biasing sequence. As a further alternative, feedforward synaptic depression could reduce input in the biasing region. After biasing below, the *I* units above the PC of T_2_ would receive more input than those below due to feedforward depression. However, those *I* units above the PC of T_2_ inhibit the *E*_*up*_ unit at the PC of T_2_, thus disadvantaging *E*_*up*_. The feedforward depression might produce some desired effects, but it requires fine-tuning and is not robust. Overall, other adaptation mechanisms as considered above might contribute to the context effects, but we expect them not to be the sole mechanism. The inclusion of such adaption mechanisms in our model would not affect its behavior, providing the facilitation of inhibition is sufficiently strong.

### Applicability and relation to other domains in neuroscience

Contextual effects on the basis of stimulus history have been described in multiple other fields of neuroscience. Since the literature is considerable, we here only discuss a few related phenomena. In audition, Raviv et al. (2012) observed an apparent attraction of the tone frequency to the mean of the prior distribution. Our model can potentially be applied to their paradigm, since their experiment also involved pitch height judgment. Preliminary simulations with a non-wrapped version of the present model indicate that its dynamics can account for these attractive effects.

In vision, bistable perception can be induced by the “apparent motion quartet,” where two pairs of points, each pair as the end points of a diagonal of an invisible rectangle, are alternately flashed and one perceives either a horizontal or a vertical motion along the edges of the rectangle. The proportion of perceived direction depends on the ratio of the length and the width of the rectangle and the perception is ambiguous when the ratio is one, i.e., the flashing dots are on a square (Hock et al., 1993). The percept can be biased by presenting lights along one pair of edges of the rectangle, suggesting a likely path connecting these points (Zhang et al., 2012). This is closely related to the present paradigm, as the visual equivalent of direction selective cells, namely motion selective cells, are likely underlying the percept, and a flash in between primes one of the two possible directions.

## Conclusions

We investigated a scenario where the perception of frequency change is stimulus history dependent. The model that we developed and analyzed here utilizes asymmetric inhibition to generate direction selectivity. The synaptic facilitation of inhibition represents a distribution of past stimuli and influences perception for future pitch change. While focused on a special set of stimuli—Shepard tones—the model readily extends to other spectrally non-periodic stimuli.

## Author contributions

Conceived the theoretical framework: CH, JR. Designed and implemented the model: CH. Wrote the paper: CH. Edited the manuscript: CH, BE, SS, JR.

### Conflict of interest statement

The authors declare that the research was conducted in the absence of any commercial or financial relationships that could be construed as a potential conflict of interest.

## Supplementary material

Audio files **“Audio1.wav,” “Audio2.wav,” “Audio3.wav,” “Audio4.wav” are stimuli examples of tritone comparison with context tones (Section The Tritone Comparison is Biased by One-sided Preceding Tones)**. In these four examples, there are 10 context tones preceding two test tones separated by a half-octave (tritone pair). In “Audio1.wav” and “Audio2.wav,” the last two tones (T_1_ and T_2_) are identical. However, listener's perception of pitch change can be opposite for these two Audios due to different spectral locations of the context tones. Most listeners would hear ascending for the last two tones in “Audio1.wav” while descending in “Audio2.wav.” Another two examples with identical T_1_ and T_2_are “Audio3.wav” and “Audio4.wav.” Context tones in “Audio1.wav” and “Audio3.wav” are in *Up bias* region (above T_1_), while those in “Audio2.wav” and “Audio4.wav” are in *Down bias* region (below T_1_).
